# BIRB796, the Inhibitor of p38 Mitogen-Activated Protein Kinase, Enhances the Efficacy of Chemotherapeutic Agents in ABCB1 Overexpression Cells

**DOI:** 10.1371/journal.pone.0054181

**Published:** 2013-01-18

**Authors:** Dan He, Xiao-qin Zhao, Xing-gui Chen, Yi Fang, Satyakam Singh, Tanaji T. Talele, Hui-juan Qiu, Yong-ju Liang, Xiao-kun Wang, Guo-qing Zhang, Zhe-sheng Chen, Li-wu Fu

**Affiliations:** 1 Department of Thoracic Surgery, Affiliated Tumor Hospital, Xinjiang Medical University, Urumuqi, China; 2 State Key Laboratory of Oncology in Southern China, Cancer Center, Sun Yat-Sen University, Guangzhou, China; 3 Department of Pharmaceutical Sciences, College of Pharmacy and Health Sciences, St. John's University, Queens, New York, United States of America; Indiana University School of Medicine, United States of America

## Abstract

ATP-binding-cassette family membrane proteins play an important role in multidrug resistance. In this study, we investigated BIRB796, an orally active inhibitor of p38 mitogen-activated protein kinase, reversed MDR induced by ABCB1, ABCG2 and ABCC1. Our results showed that BIRB796 could reverse ABCB1-mediated MDR in both the drug selected and transfected ABCB1-overexpressing cell models, but did not enhance the efficacy of substrate-chemotherapeutical agents in ABCC1 or ABCG2 overexpression cells and their parental sensitive cells. Furthermore, BIRB796 increased the intracellular accumulation of the ABCB1 substrates, such as rhodamine 123 and doxorubicin. Moreover, BIRB796 bidirectionally mediated the ATPase activity of ABCB1, stimulating at low concentration, inhibiting at high concentration. However, BIRB796 did not alter the expression of ABCB1 both at protein and mRNA level. The down-regulation of p38 by siRNA neither affected the expression of ABCB1 nor the cytotoxic effect of paclitaxel on KBV200. The binding model of BIRB796 within the large cavity of the transmembrane region of ABCB1 may form the basis for future lead optimization studies. Importantly, BIRB796 also enhanced the effect of paclitaxel on the inhibition of growth of the ABCB1-overexpressing KBV200 cell xenografts in nude mice. Overall, we conclude that BIRB796 reverses ABCB1-mediated MDR by directly inhibiting its transport function. These findings may be useful for cancer combinational therapy with BIRB796 in the clinic.

## Introduction

The multidrug resistance (MDR) which results from the overexpression of ATP-binding-cassette (ABC) family membrane proteins is one of the key reasons for cancer therapy failure which in turn might lead to mortality. So far, ABC transporters have 49 members, and they are divided into seven categories, from ABCA to ABCG [1]. Among them, ABCB1, ABCG2 and ABCCs are known as the closest proteins with multidrug resistance in cancer cells [2].

ABCB1, also named P-glycoprotein coded by *mdr1* gene, is a glycoprotein of 170-kDa, and is composed of two homologous halves, each containing six transmembrane domains and an ATP binding/utilization domain, separated by a flexible polypeptide linker. ATP binding and hydrolysis appear to be essential for the proper function of ABCB1 [3]. ABCB1 is constitutively expressed in many normal tissues including hematopoietic stem cells, natural killer cells, liver, kidney, intestinal mucosa, muscle, brain, and testis, and its functions are associated with detoxication and secretion [4]. On the other hand, ABCB1 also transports a wide range of antineoplastic drugs such as doxorubicin, vincristine, paclitaxel, and epipodophyllotoxins out of the cancer cells [5]. Increased level of ABCB1 is common in cancer cells, such as colon and kidney cancers [6]. Moreover, the expression of the ABCB1 can be induced after chemotherapy, when the tumor becomes refractory to treatment [7]. The presence of increased level of ABCB1 in several types of tumors has been correlated with poor responses to chemotherapy, short progression-free survival and overall survival [8], [9], [10]. As compared to ABCB1, ABCG2 is a half transporter that consists of six transmembrane domains and one ATP-binding site, acts as a homodimeric efflux pump, and its substrates include mitoxantrone, topotecan and SN-38, as well as fluorescent dyes such as Hoechst 33342 which is used for screening side population (SP) cell [11]. In contrast to ABCB1, ABCC1 transports a broad-spectrum of antineoplastic drugs mainly conjugated to glutathione, glucuronate and sulfate, also including vincristine and doxorubicin [12].

p38, a class of serine/threonine mitogen-activated protein (MAP) kinase, is composed of 4 isoforms (α, β, γ, and δ) with more than 60% overall sequence homology and more than 90% identity within the kinase domains. p38 is activated through phosphorylation at the Thr180-Gly-Tyr182 motif by MKK3, MKK4, and MKK6 [13]. Phosphorylated p38 activates a wide range of substrates that include transcription factors, protein kinases, and nuclear proteins, leading to diverse responses such as inflammatory responses, cell differentiation, cell-cycle arrest, apoptosis, senescence, cytokine production, and regulation of RNA splicing [14], [15]. The specific inhibitors, inactivating p38 by directly or indirectly acting on ATP-binding pocket [16], have been reported that could enhance the treatment effect of all-trans-retinoic acid in acute promyelocytic leukemia cell [17], arsenic trioxide in chronic myeloid leukemia cell [18] and bortezomib in multiple myeloma cell [19]. In addition, several evidences showed that p38 inhibitors enhanced the sensitivity of the chemotherapeutic agents in some tumor *in vivo* and *in* *vitro* [20], [21]. Therefore, we investigated the effect of the new generation p38 inhibitor BIRB796, which has entered into phase III clinical trial for the treatment of inflamation [22], on reversal of ABC transporter mediated MDR. In this report, our results showed that BIRB796 dramatically and specifically reversed the ABCB1-mediated MDR *via* inhibiting the function of ABCB1.

## Materials and Methods

### Chemicals and Agents

**Figure 1:**
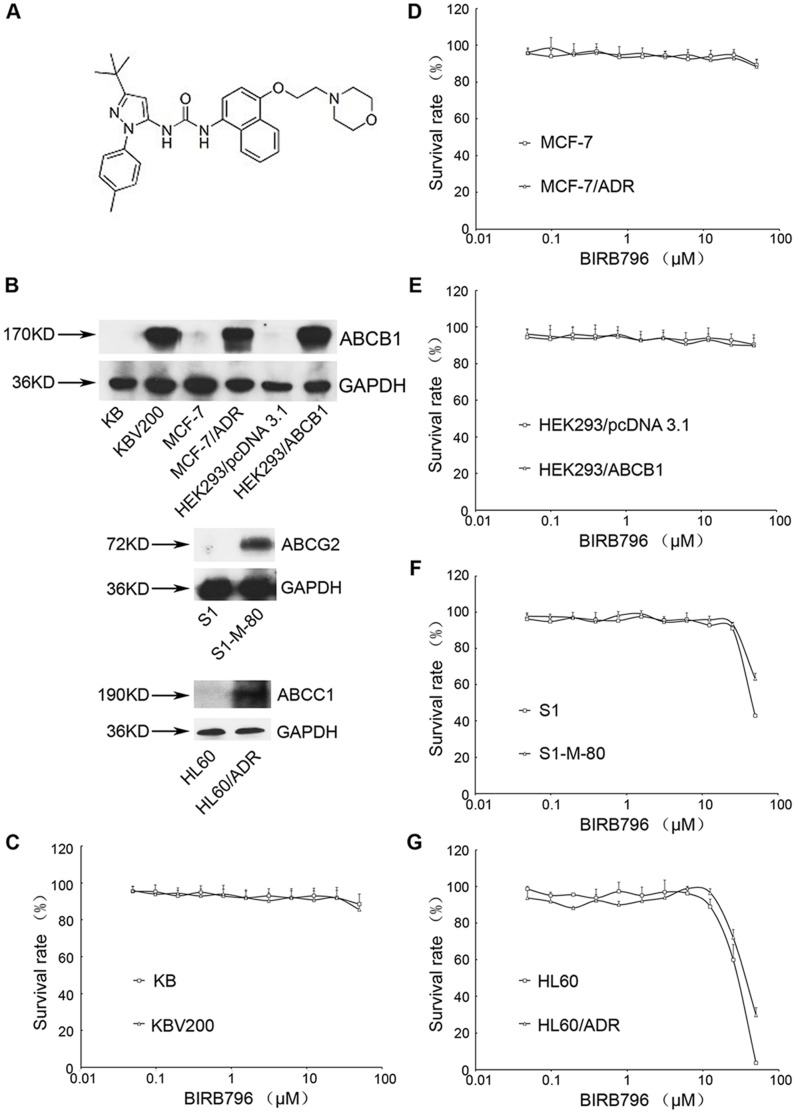
Cytotoxicity of BIRB796 in the drug-resistant and parental sensitive cancer cells. The structure of BIRB796 (A). The protein expression of ABCB1 in KB, KBV200, MCF-7, MCF-7/ADR, HEK293/pcDNA3.1 and HEK293/ABCB1; ABCG2 in S1 and S1-M1-80; ABCC1 in HL60 and HL60/ADR (B). MTT cytotoxicity assay was used to measure cell survival in KB and KBV200 (C), MCF-7 and MCF-7/ADR (D), HEK293/pcDNA3.1 and HEK293/ABCB1 (E), S1 and S1-M1-80 (F), HL60 and HL60/ADR (G) treated with BIRB796 for 72 h. Each point represents the mean ± standard deviations (SDs) for three determinations. Each experiment was performed in four replicate wells.

BIRB796 was purchased from Selleckchem, with a molecular structure shown in **Figure 1** **.** A Monoclonal antibody against ABCB1 was purchased from Santa Cruz Biotechnology (CA, USA). Glyceraldehyde-3-phosphate dehydrogenase (GAPDH) antibody was purchased from Kangchen Co. (Shanghai, China). Phospho-p38 MAP Kinase (Thr180/Tyr182) antibody, p38 MAP Kinase antibody, SignalSilence® Control siRNA (Unconjugated), SignalSilence® p38 MAP Kinase siRNA I and SignalSilence® p38 MAP Kinase siRNA II were purchased from cell signaling technology®. DMEM and RPMI-1640 were products of Gibco BRL (NY, USA). Platinum® SYBR® Green qPCR SuperMix-UDG with ROX was obtained from Invitrogen Co. Pgp-Glo™ Assay System with P-Glycoprotein was purchased from Promega Corp. Fumitremorgin C, doxorubicin, paclitaxel, 1-(4, 5-dimethylthiazol-2-yl)-3, 5-diphenylformazan (MTT), rhodamine 123, verapamil (VRP) and other chemicals were obtained from Sigma Chemical Co.

### Cell Culture

The following cell lines were cultured in DMEM or RPMI 1640 containing 10% fetal bovine serum at 37°C in the presence of 5% CO2: the human oral epidermoid carcinoma cell line KB and its vincristine-selected ABCB1-overexpressing variant KBV200 [5], the human breast carcinoma cell line MCF-7 and its doxorubicin-selected ABCB1-overexpressing variant MCF-7/ADR [23], the colon carcinoma cell line S1 and its mitoxantrone-selected ABCG2-overexpressing variant S1-M1-80 [24], [25], the human primary embryonic kidney cell line HEK293/pcDNA 3.1, and ABCB1 stable gene-transfected cell line HEK293/ABCB1 obtained from Dr. S.E. Bates (National Cancer Institute, NIH) [26], the human leukemia cell line HL60 and its doxorubicin-selected ABCC1-overexpressing variant HL60/ADR [27]. All of the transfected cells were cultured in medium with 2 mg/mL G418. All resistant cells were authenticated by comparing their fold resistance with that of the parental drug-sensitive cells and examining the expression levels of ABC transporters. All cells were grown in drug-free culture medium for >2 weeks before the assay.

### Cell Proliferation Assay

MTT assay was used to assess anti-proliferation activity [28]. Briefly, cells were seeded in 96-well plates and allowed to attach overnight. Afterward cells were preincubated with or without the BIRB796 and verapamil/fumitremorgin C (as positive control) for 1 hour and then various concentrations of chemotherapeutic drugs were added into designated wells. After 68 hours of incubation, MTT (4 mg/mL, 20 µL/well) was added to each well, and the plate was further incubated for 4 hours, allowing viable cells to change the yellow-colored MTT into dark-blue formazan crystals. Subsequently the medium was discarded, and 120 µL of dimethylsulfoxide (DMSO) was added into each well to dissolve the formazan crystals. The absorbance was determined at 655 nm by the Model 550 Microplate Reader (BIO-RAD, Hercules, CA, USA). The concentrations required to inhibit growth by 50% (IC_50_) were calculated from survival curves using the Bliss method. The degree of resistance was calculated by dividing the IC_50_ for the MDR cells by that of the parental sensitive cells. The degree of the reversal of MDR was calculated by dividing the IC_50_ for cells with the anticancer drug in the absence of BIRB796 by that obtained in the presence of BIRB796.

### Rhodamine 123 and Doxorubicin Accumulation

The intracellular rhodamine 123 and doxorubicin accumulation in ABCB1 overexpressing cells (KBV200 and MCF-7/ADR) and their parental sensitive cells (KB and MCF-7) were examined by flow cytometry. The logarithmically growing cells were treated with 2.5, 5 and 10 µM BIRB796, 10 µM verapamil as a positive control, at 37°C for 3 hours. Then rhodamine 123 (terminal concentration 5 mg/L) and doxorubicin (terminal concentration 10 µM) were added into designated wells followed by incubation for 30 min and 3 hours, respectively. The cells were then collected, centrifuged and washed twice with cold PBS. Cells were resuspended in 400 µL PBS and then analyzed by flow cytometry (Beckman Coulter, Cytomics FC500, USA).

### ABCB1 ATPase Activity Assay

The verapamil-stimulated ABCB1 ATPase activity was estimated by Pgp-Glo™ assay systems (Promega, USA). The inhibitory effect of BIRB796 was examined against verapamil-stimulated ABCB1 ATPase activity. Sodium orthovanadate (Na_3_VO_4_) was used as a selective inhibitor of ABCB1 ATPase. Following the instruction of Pgp-Glo™ Assay Systems, Pgp-Glo™ Assay Buffer, 0.25 mM Na_3_VO_4_, 0.5 mM verapamil, BIRB796 in various concentrations, 25 µg recombinant human ABCB1 membranes and 25 mM MgATP were added into designated white opaque 96-well plate (corning, USA) in turn. And then the plate were incubated at 37°C for 40 min. Subsequently, luminescence was initiated by ATP detection buffer. After incubation at room temperature for 20 min to allow luminescent signal to develop, the plate was read on a luminometer (spectraMax M5, molecular devices, USA). The changes in relative light units (△RLU) were determined by comparing Na_3_VO_4_-treated samples with BIRB796 and verapamil combination-treated samples, and thereafter, the ATP consumed was measured by comparing to a standard curve.

### Western Blot Analysis

After BIRB796 treatment, cells were harvested and rinsed twice with ice-cold PBS buffer. Cell extracts were collected in cell lysis buffer. Equal amounts of protein were resolved by SDS-PAGE and transferred onto nitrocellulose membranes. After blocked in 5% non-fat milk in TBST buffer for 2 hours at room temperature, the membranes were incubated with appropriately diluted primary antibodies overnight at 4°C. The membranes were then washed thrice with TBST buffer and incubated with HRP-conjugated secondary antibody at 1∶5000 dilution for 2 hours at room temperature. After washed thrice with TBST buffer, the protein-antibody complex were visualized by the enhanced Phototope TM-HRP Detection Kit (Cell Signaling, USA) and exposed to Kodak medical X-ray processor (Carestream Health, USA). GAPDH was used as the loading control.

### RT PCR and Real-time PCR

After concentration gradient and time gradient BIRB796 treatment, total cellular RNA was isolated by Trizol Reagent RNA extraction kit following the manufacturer’s instruction (Molecular Research Center, USA). The first strand cDNA was synthesized by RevertAid™ Premium First Strand cDNA Synthesis Kit (FERMENTAS INTERNATIONAL INC.). The PCR primers were 5′-GTGGGGCAAGTCAGTTCATT-3′ (forward) and 5′-TCTTCACCTCCAGGCTCAGT-3′ (reverse) for ABCB1; 5′-GAGTCAACGGATTTGGTCGT-3′ (forward) and 5′-GATCTCGCTCCTGGAAGATG-3′ (reverse) for GAPDH, respectively. Using the Gene Amp PCR system 9700 (PE Applied Biosystems, USA), reactions were carried out at 94°C for 2 min for initial denaturation, and then at 94°C for 30 s, 58°C for 30 s, and 72°C for 1 min. After 32 cycles of amplification, additional extensions were carried out at 72°C for 10 min. Products were resolved and examined by 1.0% agarose gel electrophoresis. Expected PCR products were 222 bp for ABCB1 and 224 bp for GAPDH, respectively.

Real-time PCR was performed by the Bio-Rad CFX96TM Real-Time (Applied Biosystems, USA). The geometric mean of the GAPDH was used as an internal control to normalize the variability in expression levels. The primers of ABCB1 and GAPDH have been described above. The PCR reactions were performed at 50°C for 2 min, 95°C for 5 min and 40 cycles at 95°C for 15 s, 60°C for 30 s. Relative quantification of ABCB1 was performed using the 2−ΔΔCt method. To ensure reproducibility of the results, all genes were tested in triplicate in three independent experiments.

### siRNA

KBV200 were seeded in 6-well plates and allowed to attach overnight. Upon discarding the medium, each well was washed thrice with PBS. Afterward 600 µL siRNA-Lipofectamine™ 2000 complexes diluted in OptiMEM® I Medium was added into designated wells followed by incubation at 37°C for 6 hours, then switched to the normal medium in each well. After 72 hours of incubation cells were harvested for MTT or western blot analysis.

### Ligand Structure Preparation

The structure of BIRB796 was built using the fragment dictionary of Maestro v9.0 and energy minimized by Macromodel program v9.7 (Schrödinger, Inc., New York, NY, 2009) using the OPLSAA force field with the steepest descent followed by truncated Newton conjugate gradient protocol. The low-energy 3D structures of BIRB796 were generated by LigPrep v2.3 and the parameters were defined based on different protonation states at physiological pH±2, and all possible tautomers and ring conformations. Ligand structures obtained from the LigPrep v2.3 run were further used for generating 100 ligand conformations for each protonated structure using the default parameters of mixed torsional/low-mode sampling function. The conformations were filtered with a maximum relative energy difference of 5 kcal/mol to exclude redundant conformers. The output conformational search (Csearch) file containing at most 100 unique conformers of BIRB796 were used as input for docking simulations into each of the drug-binding sites of human ABCB1.

### Protein Structure Preparation

The X-ray crystal structure of mouse ABCB1 in apoprotein state (PDB ID: 3G5U), in complex with inhibitors QZ59-RRR (PDB ID: 3G60), QZ59-SSS (PDB ID: 3G61) [29] and bacterial co-crystal structure of LmrA ATP-binding domain (PDB ID: 1MV5) as the template obtained from the RCSB Protein Data Bank were used to generate the homology model of human ABCB1, based on our homology modeling protocol [30], [28] The refined human ABCB1 homology model was further used to generate different receptor grids for sites 1–4 by selecting QZ59-RRR (site-1) and QZ59-SSS (site-2) bound ligands, all amino acid residues known to contribute to verapamil binding (site-3), two residues (Phe728 and Val982) known to be common to three previous sites (site-4) as previously reported by Shi et al [30], and additionally to evaluate the possibility of BIRB796 interaction at the ATP-binding site, bound ATP ligand was selected for grid generation and ensuing docking simulation of BIRB796 at ATP-binding site. In this study, we also utilized the energy minimized co-crystal structure of BIRB796-p38 MAP kinase (PDB ID: 1KV2) [31] for the purpose of comparing topological features of the allosteric binding site located adjacent to the ATP binding site of p38 MAP kinase with those present in drug/substrate-binding site of ABCB1 through residue charge surface representation. Extra precision (XP)-Glide v5.0 docking protocol was followed with the default functions (Schrödinger, Inc., New York, NY, 2009). The top scoring BIRB796 conformation at site 1 of ABCB1 was used for graphical analysis. All computations were carried out on a Dell Precision 470 n dual processor with the Linux OS (Red Hat Enterprise WS 4.0).

### Animals and Nude Mouse Xenograft Model

Athymic nude mice (BALB/c-nu/nu), 6 to 8 weeks of age and weighing 18 to 24 g, were obtained from the Center of Experimental Animals, Sun Yat-Sen University (Guangzhou, China), and were used for the KBV200 cell xenografts. All animals received sterilized food and water. All experiments were performed in accordance with the guidelines on animal care and experiments with laboratory animals (Center of Experimental Animals, Sun Yat-Sen University), which was approved by the ethics committee for animal experiments.

The KBV200-inoculated nude mouse xenograft model established by Chen et al [32] was used in this study. In brief, harvested KBV200 cells (2×10^7^ cells/0.2 mL/mouse) were implanted subcutaneously under the shoulder in the nude mice. When the tumors reached a mean diameter of 0.5 cm^3^, the mice were divided randomly into four groups and were treated with the following regimens: 1) saline solution (every 3 days×5); 2) paclitaxel (18 mg/kg i.p., every 3 days×5); 3) BIRB796 (10 mg/kg p.o., every 3 days×5) [33]; and 4) paclitaxel (18 mg/kg i.p., every 3 days×5) plus BIRB796 (10 mg/kg p.o., every 3 days×5, administered 1 h before injection of paclitaxel). The body weights of the animals and the two perpendicular tumor diameters (A and B) were recorded every 3 days, and the tumor volume (V) was estimated according to the following formula: V = (π/6)[(A+B)/2]^3^. The curves for tumor growth and body weight were drawn according to tumor volume and time of implantation. The mice were anesthetized and killed when the mean tumor weight in the control group was more than 1 g. Tumor tissues were excised from the mice, and their weights were measured. The ratio of growth inhibition (IR) was calculated according to the following formula: IR = [1– (mean tumor weight for experimental group/mean tumor weight for control group)] ×100%.

### Statistical Analysis

All experiments were repeated at least three times. Microsoft Office Excel 2010 and the statistical software SPSS13.0 were used in data processing and analyzing the significance using the student’s t-test. All P-values were two-sided, and the statistical significance was determined at *P*<0.05.

## Results

### BIRB796 Enhances the Efficacy of Chemotherapeutic Agent in ABCB1 Overexpressing Cells but not in ABCC1 and ABCG2 Overexpressing Cells

Firstly, ABCB1 was overexpressed in KBV200, MCF-7/ADR and HEK293/ABCB1 cells, while ABCG2 and ABCC1 were overexpressed in S1-M-80 and HL60/ADR cells, respectively (**Figure 1** **. B**). The basal expressions of ABCB1, ABCC1 and ABCG2 in the parental cell lines were nearly undetectable. MTT assay showed that five MDR cells exerted much higher tolerance to multiple chemotherapeutic agents than their parental cell lines. The mean IC_50_ values of chemotherapeutic agents were shown in **Table 1** and **Table 2**.

**Table 1:** Effect of BIRB796 on reversing ABCB1-mediated drug resistance.

IC_50_± SD (µM) (fold-reversal)				
Compounds	KB		KBV200	
Doxorubicin	0.0127±0.0021		2.9660±0.0607	
+2.5 µM BIRB796	0.0122±0.0020	(1.04)	0.5507±0.0793	(5.39)^b^
+5 µM BIRB796	0.0148±0.0006	(0.86)	0.1143±0.0200	(25.95)^b^
+10 µM BIRB796	0.0124±0.0021	(1.02)	0.0874±0.0100	(33.94)^b^
+10 µM verapamil	0.0141±0.0010	(0.90)	0.0504±0.0069	(58.85)^b^
Paclitaxel	0.0024±0.0007		0.2650±0.0021	
+2.5 µM BIRB796	0.0023±0.0019	(1.04)	0.0990±0.0042	(2.68)^b^
+5 µM BIRB796	0.0025±0.0019	(0.96)	0.0530±0.0021	(5.00)^b^
+10 µM BIRB796	0.0022±0.0013	(1.09)	0.0150±0.0007	(17.67)^b^
+10 µM verapamil	0.0022±0.0010	(1.09)	0.0080±0.0007	(33.13)^b^
Cisplatin	1.1806±0.0192		8.5635±0.1801	
+10 µM BIRB796	1.3018±0.2155	(0.91)	6.7960±0.1258	(1.26)
+10 µM verapamil	1.0408±0.0346	(1.13)	7.5878±0.5150	(1.13)
	MCF-7		MCF-7/ADR	
Doxorubicin	0.1897±0.0584		19.0895±5.2192	
+2.5 µM BIRB796	0.1747±0.0370	(1.09)	3.7570±0.1146	(5.08)^b^
+5 µM BIRB796	0.1570±0.0546	(1.21)	2.1485±0.8591	(8.89)^b^
+10 µM BIRB796	0.1691±0.0538	(1.12)	1.0695±0.6300	(17.85)^b^
+10 µM verapamil	0.2135±0.0219	(0.89)	0.4750±0.1655	(40.19)^b^
Paclitaxel	0.0056±0.0025		1.2285±0.1520	
+2.5 µM BIRB796	0.0043±0.0023	(1.30)	0.4680±0.1075	(2.63)^b^
+5 µM BIRB796	0.0050±0.0026	(1.12)	0.3835±0.1718	(3.20)^b^
+10 µM BIRB796	0.0047±0.0031	(1.19)	0.0439±0.0054	(27.98)^b^
+10 µM verapamil	0.0049±0.0020	(1.14)	0.0416±0.0023	(29.53)^b^
Cisplatin	5.7035±0.1168		51.8083±3.4921	
+10 µM BIRB796	5.4934±0.3039	(1.04)	59.7638±5.8154	(0.87)
+10 µM verapamil	5.4462±0.3209	(1.05)	45.9217±1.9789	(1.13)
	HEK293/pcDNA3.1		HEK293/ABCB1	
Doxorubicin	0.1818±0.0153		3.4242±0.0421	
+2.5 µM BIRB796	0.1652±0.0068	(1.10)	1.8753±1.5185	(1.83)^a^
+5 µM BIRB796	0.1765±0.0049	(1.03)	1.2899±0.8501	(2.65)^b^
+10 µM BIRB796	0.1690±0.0127	(1.08)	0.4103±0.0349	(8.35)^b^
+10 µM verapamil	0.1646±0.0161	(1.10)	0.2480±0.0085	(13.81)^b^

Note: cell survival was determined by MTT assay as described in “Materials and Methods”. Data are the means ± SD of at least three independent experiments performed in triplicate. The fold-reversal of MDR was calculated by dividing the IC_50_ for cells with the anticancer drug in the absence of BIRB796 or by that obtained in the presence of BIRB796 or verapamil. a and b represent *P*<0.05 and *P*<0.01 respectively, for values versus that obtained in the absence of BIRB796.

**Table 2:** Effect of BIRB796 on reversing ABCG2- and ABCC1 -mediated drug resistance.

IC_50_± SD (µM) (fold-reversal)				
Compounds	S1		S1-M-80	
Topotecan	0.2617±0.0545		15.9287±2.0380	
+10 µMBIRB796	0.2930±0.0400	(0.89)	14.7547±2.6494	(1.08)
+2.5 µMfumitremorgin C	0.2594±0.0939	(1.01)	0.9067±0.1847	(17.57)^a^
	HL60		HL60/ADR	
Doxorubicin	0.0063±0.0009		7.4348±0.6185	
+10 µMBIRB796	0.0068±0.0008	(0.93)	6.8734±0.1569	(1.08)
+10 µMverapamil	0.0062±0.0006	(1.02)	0.7752±0.2661	(9.59)^a^

Note: cell survival was determined by MTT assay as described in “Materials and Methods”. Data are the means ± SD of at least three independent experiments performed in triplicate. The fold-reversal of MDR was calculated by dividing the IC_50_ for cells with the anticancer drug in the absence of inhibitor by that obtained in the presence of inhibitor. a represent *P*<0.01, for values versus that obtained in the absence of inhibitor.

The intrinsic *in vitro* toxicity of BIRB796 on different cells was examined by the MTT assay. Notably, the results showed that BIRB796, at 10 µM, had no obvious cytotoxic effect to all cell lines, and more than 90% cells were survived (**Figure 1** **. C, D, E, F and G**). The cytotoxic effect of chemotherapeutic agents in KB, KBV200, MCF-7, MCF-7/ADR, HEK293/pcDNA3.1 and HEK293/ABCB1 was tested in the presence of 2.5, 5 and 10 µM BIRB796 and 10 µM verapamil as a positive control. The mean IC_50_ values of chemotherapeutic agents in various pairs of the sensitive and resistant cells at different concentrations of BIRB796 were shown in **Table 1**. In ABCB1-overexpressing cells, BIRB796 significantly enhanced the cytotoxic effect of doxorubicin (5.39, 25.95 and 33.95-reversing fold) and paclitaxel (2.68, 5 and 17.67-reversing fold) in KBV200; doxorubicin (5.08, 8.89 and 17.85-reversing fold) and paclitaxel (2.63, 3.20 and 27.98-reversing fold) in MCF-7/ADR (*P*<0.05) in a concentration-dependent manner. Meanwhile the similar result was observed in transfected cell HEK293/ABCB1 (1.83, 2.65 and 8.35-reversing fold to doxorubicin). Nevertheless, BIRB796 did not change the cytotoxic effect of chemotherapeutic agents on the parental cells KB, MCF-7 and HEK293/pcDNA3.1 to doxorubicin and paclitaxel. In addition, BIRB796 didn’t change the cytotoxic effect of cisplatin which is not the substrate of ABCB1 in both drug-resistant- (KBV200 and MCF-7/ADR) and the parental- (KB and MCF-7) cells.

Moreover, we tested the cytotoxic effect of topotecan in ABCG2-overexpressing S1-M-80 cell and that of doxorubicin in ABCC1-overexpressing HL60/ADR cell in the presence of 10 µM BIRB796, respectively. The result showed that BIRB796 had no reversal effect in ABCG2- and ABCC1-overexpressing cells (**Table 2**).

### BIRB796 Increases the Intracellular Accumulation of Rhodamine 123 and Doxorubicin in ABCB1-Overexpressing Cells

The data above indicated that BIRB796 specifically sensitized the ABCB1-overexpressing cells to several chemotherapeutic agents which are the substrates of ABCB1. To understand the underlying mechanism, we determined the intracellular accumulation of rhodamine 123 and doxorubicin in drug-resistant cells and their parental cells by flow cytometric analysis.

**Figure 2:**
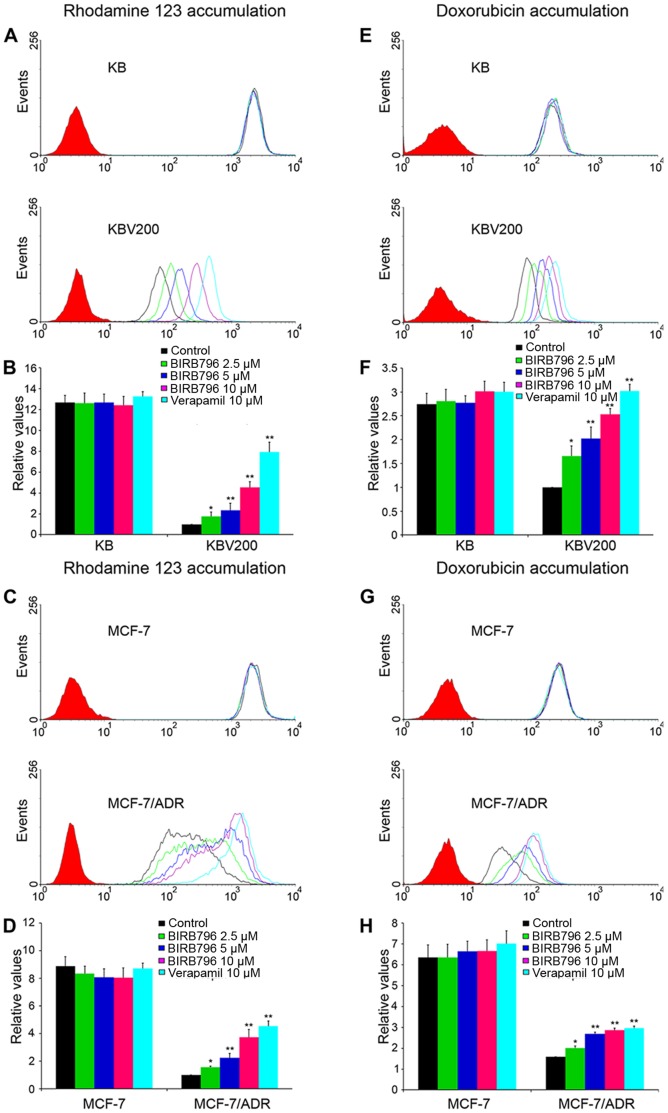
Effect of BIRB796 on the accumulation of rhodamine 123 and doxorubicin. The accumulation of rhodamine 123 in KB, KBV200 (A, B) and in MCF-7, MCF-7/ADR (C, D). The accumulation of doxorubicin in KB, KBV200 (E, F) and MCF-7, MCF-7/ADR (G, H) was measured by flow cytometric analysis as described in “materials and methods”; and the histogram (B, D, F and H), presented as fold change in fluorescence intensity relative to control MDR cells, was the result in two pair of cells, respectively. Columns, means of triplicate determinations; bars, SDs. *, *P*<0.05; **, *P*<0.01, versus the MDR control group, respectively.

Firstly, the intracellular accumulation of rhodamine 123 and doxorubicin were significantly higher in KB (12.67 and 2.74-fold) and MCF-7 (8.89 and 6.36-fold) than that in KBV200 and MCF-7/ADR (**Figure 2**). When the drug-resistant cells were treated in the presence of BIRB796 at 2.5, 5 and 10 µM, the intracellular accumulation of rhodamine 123 was 1.77, 2.34 and 4.53-fold higher than that in untreated KBV200; and 1.65, 2.02 and 2.53-fold higher than that in untreated MCF-7/ADR, respectively (**Figure 2** **. B and D**). Meanwhile, the intracellular accumulation of doxorubicin was 1.56, 2.26 and 3.74-fold higher than that in untreated KBV200; and 1.59,2.01 and 2.86-fold higher than that in untreated MCF-7/ADR (**Figure 2** **. F and H**). However, no significant change in the intracellular accumulation of rhodamine 123 and doxorubicin were observed at different concentrations of BIRB796 in KB and MCF-7 as compared to untreated KB and MCF-7. Taken together, these data suggest that BIRB796 was able to regulate ABCB1 mediated transport in MDR cells.

### BIRB796 Bidirectionally Mediates the ATPase Activity of ABCB1

**Figure 3:**
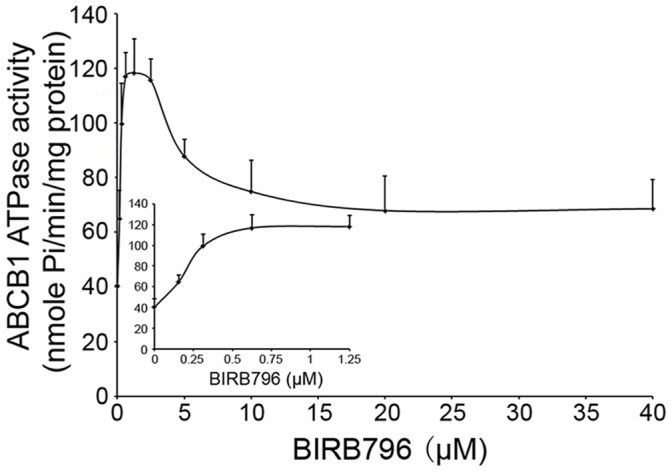
Effect of BIRB796 modulating the activity of ABCB1 ATPase. The Vi-sensitive ATPase activity of ABCB1 in membrane vesicles was determined with different concentrations of BIRB796 as described in the "Materials and Methods" section. Each point represents the mean ± SDs for triplated independent determinations.

The drug-efflux function of ABC transporter depends on the ATP hydrolysis that reflects ATPase activity. Therefore, we tested the modulatory effect of BIRB796 on vanadate-sensitive ATPase activity in recombinant human ABCB1 membranes. As illustrated in **Figure 3**, BIRB796 bidirectionally modulated the ATPase activity of ABCB1, stimulating at low concentration, and inhibiting at high concentration. The peak of the ATPase activity was at 1.25 µM; then the ATPase activity was inhibited dose-dependently at 2.5, 5 and 10 µM. Therefore, we suggest that BIRB796 reverses MDR in ABCB1-overexpressing cells by inhibiting the ATPase activity and may act as a substrate of ABCB1.

### BIRB796 does not Affect the Expression of ABCB1 both at Protein and mRNA Level

**Figure 4:**
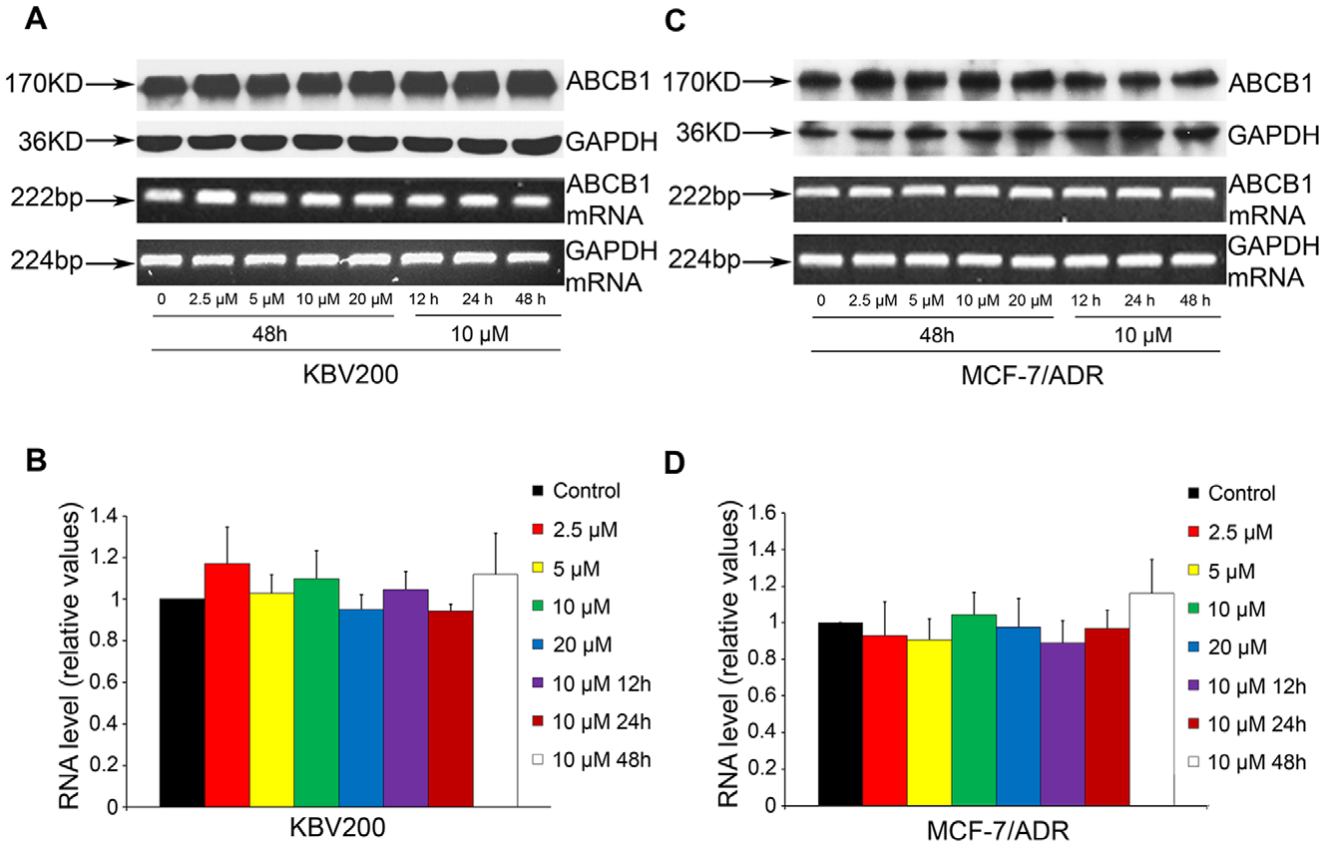
Effect of BIRB796 on the expression of ABCB1 in MDR cells. KBV200 (A and B) and MCF-7/ADR (C and D) were treated with BIRB796 at various concentrations for 72 h and for various time at 10 µM. Equal amounts of total cell lysates were loaded and detected by Western blot. The mRNA level of ABCB1 was determined by PCR and RT-PCR as described in “Materials and Methods”; A representative result were shown from at least three independent experiments.

Either decreasing the expression or inhibiting the function of ABCB1 can affect the reversal of ABC transporter-mediated MDR. Hence, we determined the expression of ABCB1 at the protein and mRNA level upon treatment with BIRB796 by using Western blot and RT-PCR analyses, respectively. Our results showed that after incubation with BIRB796, at both concentration- and time-gradient, no remarkable difference was observed in KBV200 (**Figure 4** **. A and B**) and MCF-7/ADR cells (**Figure 4** **. C and D**). These data indicated that BIRB796 mediated the reversal of MDR by inhibiting the function of ABCB1, and not due to inhibition of ABCB1 expression.

### Down-regulation of p38 does not Affect the Expression of ABCB1 and the Cytotoxic Effect of Chemotherapeutic Agents in Drug Selected Cells

**Figure 5:**
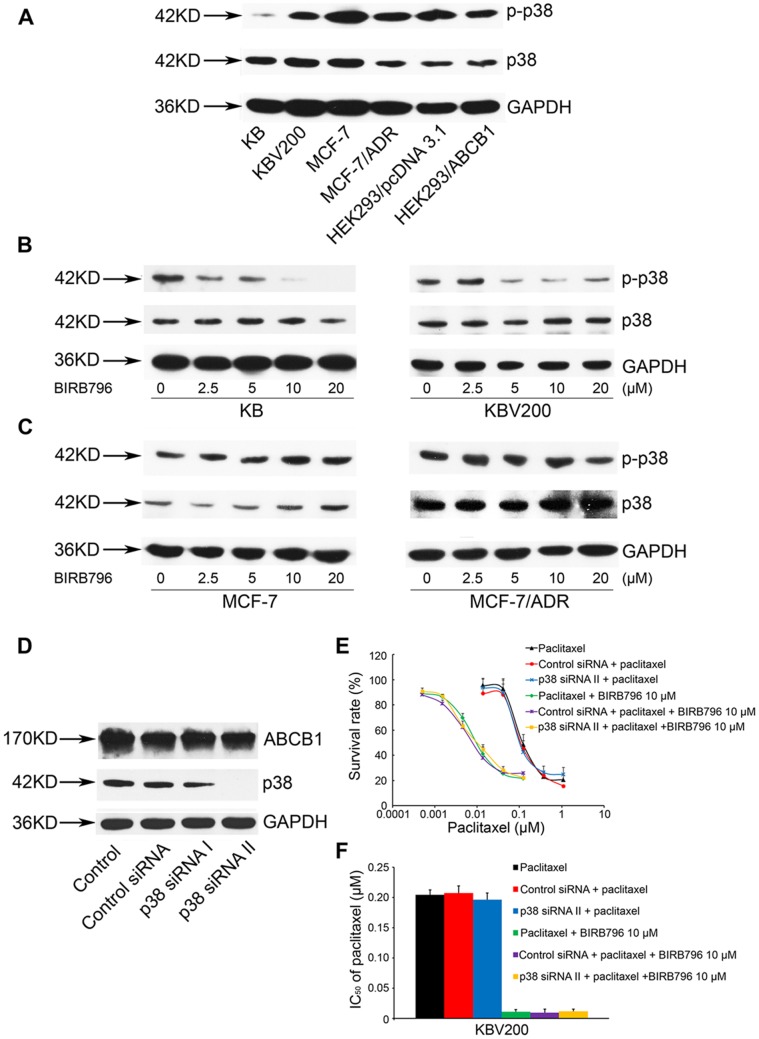
Effect of modulating ABCB1 with silencing p38 by siRNA. The basal expression of phosphorylation of p38 was tested in KB, KBV200, MCF-7, MCF-7/ADR, HEK293/pcDNA3.1 and HEK293/ABCB1 (A). The inhibitory effect of phosphorylated p38 in KB, KBV200 (B) and in MCF-7, MCF-7/ADR (C) treated with BIRB796 at various concentrations after 24 h. The expression of ABCB1 in KBV200 transfected with Control siRNA, p38 siRNA I and p38 siRNA II after 72 h (D). MTT assay was used to draw the survival rate curve (E) and the IC_50_ histogram (F) of paclitaxel after silencing p38 by p38 siRNA II. Each point represents the mean ± standard deviations (SDs) for three determinations. Columns, means of triplicate determinations; bars, SDs.

Earlier study reported that the p38 was highly expressed in some specific drug-resistant tumor cells [20], [34], [21]. To investigate whether BIRB796, at reversal concentration, could affect the p38 signaling pathway, we tested basal expression of p38 in KB, KBV200, MCF-7, MCF-7/ADR, HEK293/pcDNA3.1 and HEK293/ABCB1. As shown in the **Figure 5** **. A**, phosphorylated p38 was observed in three pairs of cells. Then, we found the dramatic inhibition of p38 expression at reversing concentrations of BIRB796 in KB and KBV200 but not in MCF-7 and MCF-7/ADR (**Figure 5** **. B and C)**. Therefore, we chose KBV200, which had the higher expression of p-p38 than KB, to down-regulate the expression of p38 by p38 siRNA II, followed by MTT to test the cytotoxic effect of paclitaxel. The result showed that down-regulation of p38 neither changed the expression of ABCB1 (**Figure 5** **. D**) nor the IC50 of paclitaxel in KBV200 (**Figure 5** **. E and F**). Thus we concluded that the p38 signaling pathway is not involved in the regulation of ABCB1. The reversing effect of BIRB796 on ABCB1-mediated MDR is a result of its direct action on ABCB1 rather than through p38 signaling pathway.

### BIRB796 Docking Analysis with Human ABCB1 Homology Model

**Figure 6:**
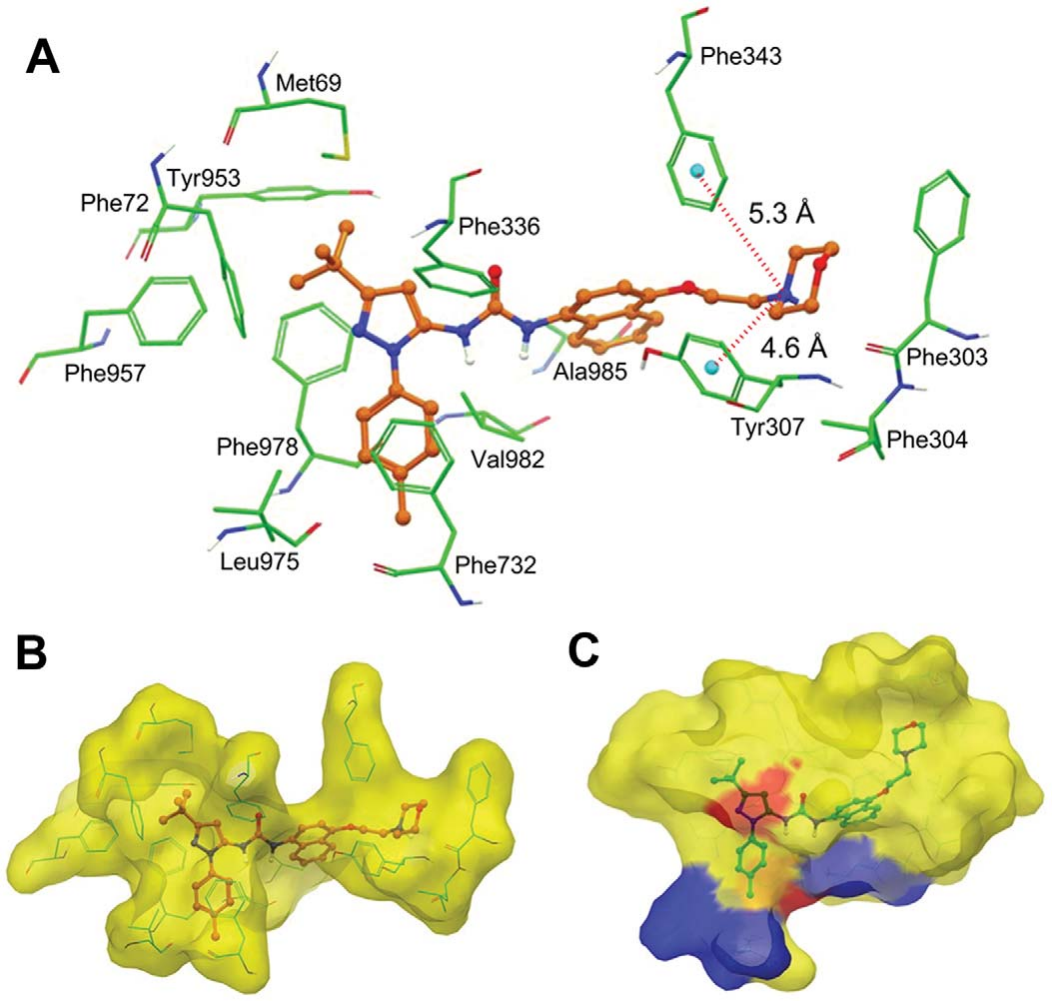
XP-Glide predicted binding mode of BIRB796 with homology modeled ABCB1. A, Binding mode of BIRB796 within the drug binding site-1 of human ABCB1. Important amino acids are depicted as sticks with the atoms colored as carbon – green, hydrogen – white, nitrogen – blue, oxygen – red, sulfur – yellow, whereas the BIRB796 is shown as ball and stick model with the same color scheme as above except carbon atoms are represented in orange. Dotted red line indicates distances. Human ABCB1 (B) and p38 MAP kinase (C) are represented as Macromodel surface as per the residue charge (electropositive charge: blue, electronegative charge: red, neutral (hydrophobic): yellow) as implemented in Maestro. Docked conformation of BIRB796 within the drug binding site-1 of human ABCB1 and bound BIRB796 within the allosteric site of p38 MAP kinase is also shown as ball and stick model.

In the absence of the crystal structure of ABCB1, we developed human ABCB1 homology model based on the crystal structure of mouse ABCB1 [29]. To understand the binding mechanism of BIRB796 to the homology model of human ABCB1 at a molecular level, docking studies were performed on all the possible binding sites as described in materials and methods section. The binding energy data for the docked conformations of BIRB796 at site-1(−9.21 kcal/mol), site-2(−7.96 kcal/mol), site-3(−5.59 kcal/mol), site-4(−8.77 kcal/mol) and ATP-binding site(−2.83 kcal/mol) clearly indicated the QZ59-*RRR* binding site of ABCB1 *i.e*., site-1, as the most favorable site for BIRB796 binding. Therefore the binding model of BRB796 at the site-1 of ABCB1 is discussed below and shown in **Figure 6** **. A**. The tolyl substituent on the pyrazole ring is involved in π- π stacking interaction with the phenyl side chain of Phe732 and hydrophobic contacts with the side chains of Leu975 and Phe978. The pyrazole ring is stabilized through aromatic-aromatic type of interactions with the side chains of Phe978 and Phe336. The *tert*-butyl substituent on the pyrazole ring is located within a large hydrophobic cavity formed by the side chains of Met69, Phe72, Tyr953, Phe957 and Phe978. The urea functional group is found to be not in direct contact with any of the residues which may suggest its role in correct positioning of other important features of BIRB796. The naphthalene ring is stabilized through hydrophobic interactions with Phe343, Val982, and Ala985. The protonated nitrogen atom of the morpholine ring is located at a distance of ∼5 Å from the centroids of the phenyl rings of both Tyr307 and Phe343 thus may involve in favorable cation-π type of interaction. The hydrophobic portion of the ethoxymorpholine substituent is in hydrophobic contacts with the side chains of Phe303, Leu304, Tyr307 and Phe343. Thus hydrophobic interactions contribute most to the binding affinity of BIRB796 towards ABCB1 drug-binding site-1.

### BIRB796 Reverses ABCB1-mediated MDR in the Nude Mouse Xenograft Model

**Figure 7:**
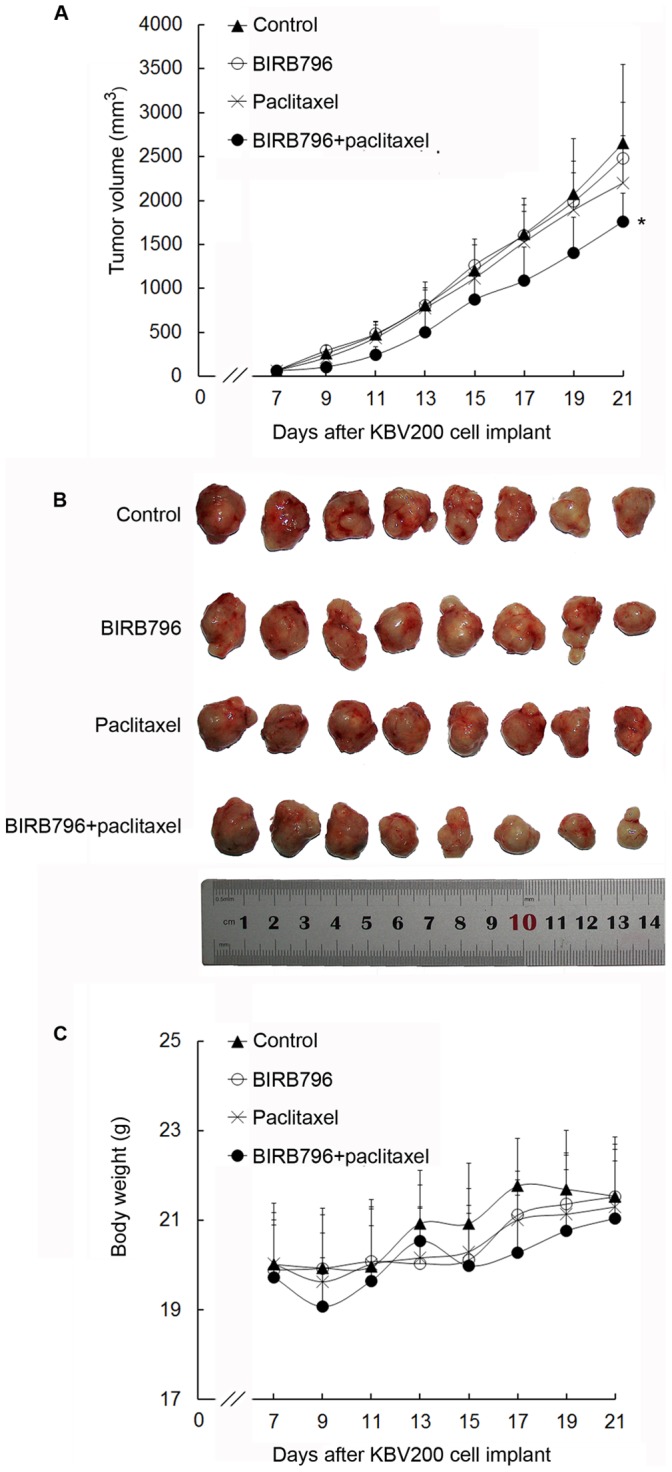
Potentiation of the antitumor effects of paclitaxel by BIRB796 in KBV200 xenograft model. KBv200 cells were collected and implanted into the mice for the chemotherapeutic studies. After 6 days, when the subcutaneous tumors were approximately 0.5×0.5 cm^2^ (perpendicular diameters) in size, mice were divided randomly into the following four treatment groups: control (saline solution), BIRB796 (10 mg/kg p.o., every 3 days×5), paclitaxel (18 mg/kg i.p., every 3 days×5), and paclitaxel (18 mg/kg i.p., every 3 days×5) plus BIRB796 (10 mg/kg p.o., every 3 days×5, administered 1 h before paclitaxel administration). A, the tumor growth curve was drawn according to tumor volume and time of implantation. B, tumor tissues were excised from the mice and their weights were measured. C, animals’ body weights were measured every 2 days, for modulation of the drug dosage. The mice were anesthetized and killed when the mean tumor weight in the control group was more than 1 g. *, *P*<0.05.

We have shown that BIRB796 could reverse the ABCB1-mediated MDR *in vitro*,however, its reversal effect *in vivo* is still unknown. Therefore, we used KBV200 to establish MDR human carcinoma xenografts model in Athymic nude mice (BALB/c-nu/nu). As the **Table 3** showed, the mean xenograft weigh of BIRB796 plus paclitaxel group(1.14±0.48 gram) was lighter than control, BIRB796 or paclitaxel group(1.84±0.61, 1.80±0.62 and 1.65±0.29 gram)(*P*<0.05). The inhibition rate of BIRB796, paclitaxel and BIRB796 plus paclitaxel was 1.93%, 9.93% and 38%. There was no significant difference in xenograft weigh between animals treated with saline, BIRB796, or paclitaxel, which indicated that single BIRB796 had no antineoplastic effect *in vivo*; and KBV200 xenografts were resistant to paclitaxel. Importantly, the combination of BIRB796 and paclitaxel produced a significant inhibition of tumor growth, compared with control, paclitaxel or BIRB796 alone (**Figure 7** **, A and B**). Furthermore, at the doses tested, no death or apparent decrease in body weight was observed in the combination treatment groups, which suggests that the combination regimen did not increase the incidence of toxic side effects (**Figure 7** **, C**).

**Table 3:** Tumor growth change before and after treatment (mean ± SD).

Group	Animal per group		Average body weight (g)		Mean tumor volume(mm^3^)		Mean xenograftweigh (g)	IR(%)
	Startday 7	Endday 21	Startday 7	Endday 21	Startday 7	Endday 21		
Control	8	8	20.03±1.35	21.3±1.28	64.68±15.94	2655.66±894.61	1.84±0.61	–
BIRB796	8	8	19.89±1.28	21.53±1.17	65.92±15.39	2483.69±636.38	1.80±0.62	1.93
Paclitaxel	8	8	20.02±0.99	21.54±1.33	63.52±13.39	2201.43±533.59	1.65±0.29	9.93
BIRB796+Paclitaxel	8	8	19.73±1.17	21.04±1.29	62.84±11.50	1764.16±320.84	1.14±0.48^*^	38.00

Note: *, *P*<0.05. *t*-test was used to analyse the differences of xenograft weigh between the BIRB796 plus paclitaxel group and other three groups(control, BIRB796 or paclitaxel).

## Discussion

In the past 30 years, significant efforts have been made to search and design the specific ABCB1 inhibitors and this has resulted in the development of three generations of ABCB1 inhibitors. However, up to now, none of the compounds from the three generations have been approved for clinical use [35], [36], [37]. Mounting evidences show several tyrosine kinase inhibitors (TKIs), including lapatinib [23], neratinib [28], erlotinib [38], cediranib [39], vandetanib [40] and axitinib [41] can reverse MDR to antineoplastic drugs mediated by ABC transporters. However, the reversal potential of these TKIs has not been determined in clinical trials. Consequently, it is still necessary to develop more efficacious, non-toxic and less expensive compounds to reverse MDR in cancer cells.

In this study, we investigated the reversing effect of BIRB796 on ABCB1-, ABCG2- and ABCC1-overexpressing cells. The result showed that BIRB796 sensitized ABCB1-overexpressing cells to doxorubicin and paclitaxel. But for cisplatin which is not the substrate of ABCB1, BIRB796 has no reversing effect both in resistant and sensitive cells. In addition, BIRB796 had no significant effect on sensitivity of ABCG2 and ABCC1-overexpressing cells to their substrate. These data indicated that the reversing ability of BIRB796 was specific to ABCB1. The assay of rhodamine 123 and doxorubicin accumulation further ascertained this result. Since Western blot and realtime PCR analyses showed no effect on the expression of ABCB1 by BIRB796 both at protein and mRNA level, we focused on the ATPase activity of ABCB1. As a result, BIRB796 bidirectionally modulated the ATPase activity of ABCB1, which is similar to vinblastine, verapamil, and paclitaxel stimulating ATPase activity at low concentrations but inhibiting the activity at high concentrations [3]. Therefore, BIRB796 might potentially be a substrate of ABCB1.

Molecular interaction between human ABCB1 and BIRB796 reveals some important pharmacophoric features as discussed below. The pharmacophoric features such as hydrophobic groups and/or aromatic ring centers, and positively charged ionizable group (tertiary amine) that are required for ABCB1 inhibition [42] are present in the structure of BIRB796. Further ligand-based studies suggested the existence of an underlying correlation between ABCB1 inhibitory activity and lipophilicity of the compounds [43], [44], [45]. In this manner, BIRB796 exhibited a QikProp v3.2 derived ClogP_o/w_ value of 5.65 which is indicative of it’s highly lipophilic nature. Further, in **Figure 6** **B** and **C**, it clearly indicates that the BIRB796 binding sites on both the targets share a significant hydrophobic component, supporting the observed affinity of BIRB796 to human ABCB1. Taken together, understanding the nature of the molecular interactions gained through these molecular modeling studies provide structural clues to optimize the activity of BIRB796 towards ABCB1.

The role of p38 signaling pathway in cancer drug resistance is still controversial and depends on the cell type [46], [34]. Our data (**Figure 5** **, B and C**) aims to investigate whether BIRB796, at reversal concentration, could affect the p38 signaling pathway. In KB and KBV200 cells, BIRB796 inhibited p-p38 at 5 µM. Nevertheless, down-regulation of p38 by siRNA could not reproduce the reversal effect of BIRB796 against paclitaxel-induced growth inhibition. Therefore, we rule out the affection of p38 signaling pathway against ABCB1-mediated MDR in KBV200 cell. Meanwhile, in MCF-7 and MCF-7/ADR cells, BIRB796 did not affect p-p38 expression even at 20 µM. We conclude the reason might be the sensitivity against inhibition of p-p38 by BIRB796 in different cell types used. The higher concentration of BIRB796 might inhibit the p-p38 in MCF-7 and MCF-7/ADR. Importantly, however, we had shown that BIRB796 at 10 µM could reverse the ABCB1-induced MDR (**Table 1**). Therefore, we concluded that the reversal effect of BIRB796, rather than p38 signaling pathway, was due to pharmacological inhibtion of the function of ABCB1.

Moreover, our result showed that BIRB796 could also reverse ABCB1-mediated MDR *in vivo*, and did not enhance the toxicity of paclitaxel in nude mice. In phase I clinical trial of human endotoxemia, BIRB796 not only inhibited the proinflammatory cytokines but also reduced LPS-induced clinical signs and symptoms; the plasma level of BIRB796 in high-dose group(600 mg)was 7.38±1.64 µM [47], which has reached the median reversal concentration in our study. In phase III clinical trial of Crohn’s disease, the treatment of BIRB796 with long-term continuous uptake (2 months) were well tolerated. And the main side effect is asymptomatic elevation of liver enzymes within the 2-fold upper limit of normal [22]. Since chemotherapy is intermittent, it is still worth evaluating the therapeutic effect and toxicity of chemotherapy combination with BIRB796 in clinic.

In conclusion, this study provides the first *in vitro* and *in vivo* evidence that BIRB796 significantly enhances the efficacy of chemotherapeutic drugs in ABCB1-overexpressing MDR cells, which is achieved by inhibiting ABCB1 ATPase activity and function. The MDR reversal seems to be independent of p38 signaling pathway. This interaction of BIRB796 with the drug transporter may affect treatment outcome of combination of BIRB796 and conventional chemotherapeutic drugs.
